# Evaluation of a thermostable Newcastle disease virus strain TS09-C as an in-ovo vaccine for chickens

**DOI:** 10.1371/journal.pone.0172812

**Published:** 2017-02-24

**Authors:** Guoyuan Wen, Lintao Li, Qingzhong Yu, Hongling Wang, Qingping Luo, Tengfei Zhang, Rongrong Zhang, Wanpo Zhang, Huabin Shao

**Affiliations:** 1 Institute of Animal Husbandry and Veterinary Sciences, Hubei Academy of Agricultural Sciences, Wuhan, China; 2 Hubei Key Laboratory of Animal Embryo and Molecular Breeding, Wuhan, China; 3 Key Laboratory of Prevention and Control Agents for Animal Bacteriosis (Ministry of Agriculture), Wuhan, China; 4 College of Veterinary Medicine, Huazhong Agricultural University, Wuhan, China; 5 US National Poultry Research Center, Agricultural Research Services, United States Department of Agriculture, Southeast Poultry Research Laboratory, Athens, Georgia, United States of America; Sun Yat-Sen University, CHINA

## Abstract

In-ovo vaccination is an attractive immunization approach for poultry industry. However, most of the Newcastle disease virus (NDV) vaccine strains used after hatch are unsafe, as in-ovo vaccines, due to their high pathogenicity for chicken embryos. In this study, we evaluated the safety and immunogenicity of a thermostable NDV strain TS09-C, derived from V4 strain, as in-ovo vaccine. Chickens in-ovo vaccinated with the parental V4 strain displayed greatly reduced hatchability and severe histopathological lesions in both trachea and intestine tissues, while the hatchability was not affected by in-ovo vaccination withTS09-C strain. The safe dose that infected all chicken embryos without obviously histopathological lesions was 10^3.0^ EID_50_ per bird. In-ovo vaccination of chickens with TS09-C virus conferred complete protection against virulent NDV challenge. Results suggest that the thermostable NDV strain TS09-C is a safe and immunogenic in-ovo vaccine candidate that can be delivered quickly and uniformly, and induce earlier immune response.

## Introduction

Newcastle disease (ND) is one of the most important infectious diseases of poultry. Newcastle disease virus (NDV), the causative agent of ND, is classified into lentogenic, mesogenic, and velogenic strains based on the pathogenicity for chickens[1]. Among three pathotypes, velogenic NDV can produce severe neurological and respiratory signs, as well as suboptimal egg production and egg quality, and poses considerable threat to the poultry industry worldwide[2]. Vaccination with live vaccines is a common strategy for ND control in many countries[3]. Several approaches of vaccine administration have been employed in the poultry industry. These methods include drinking water, eye-drop, and aerosol administration.

The automated machines have already been developed to immunize chickens via direct injection into chicken embryos (in-ovo vaccination)[4]. Up to 50,000 eggs per hour can be vaccinated using this method[5]. Currently, over 80% of US broilers are in-ovo vaccinated with Marek’s disease vaccine[6]. For NDV, the commercial live vaccines used after hatch are not safe for in-ovo vaccination, and can cause death of chicken embryos[7]. A complex of live vaccine strain Hitchner B1 and NDV neutralizing antibody has been developed as in-ovo vaccine[8], but such vaccines are difficult to standardize. In Europe, a live in-ovo vaccine NDW, sub-strain of Ulster 2C strain, was licensed but not marketed, because a safe dose of NDW that infected all the embryos without killing some of them has not been established[9]. A recombinant attenuated avian influenza virus (AIV) expressing both hemagglutinin gene of H5 AIV and hemagglutinin-neuraminidase gene of NDV, was generated and evaluated [10]. This recombinant virus, when used as in-ovo vaccine, could confer90% and 80% protection against H5 AIV and NDV challenge, respectively, as early as 3 weeks post-hatch[10]. However, the effect of this recombinant virus on hatchability was not addressed in detail. Therefore, there is a real need to search for a safe and immunogenic in-ovo NDV vaccine for control of this disease.

Thermostable NDV vaccine strains, such as V4 and I-2, have been used widely to protect village chickens against ND, due to their decreased dependence on cold chain for transport and storage[11]. Previously, we have developed a new thermostable NDV stain TS09-C by serial passages of strain V4 in BHK-21 cells[12,13]. In the present study, we evaluated the safety and efficacy of NDV strain TS09-C as an in-ovo vaccine against virulent NDV challenge.

## Materials and methods

### Animals and viruses

Specific-pathogen-free (SPF) Leghorn chicken embryos were purchased from Merial-Vital, Beijing, China. Chickens were hatched in a contained environment at 37.5°C and humidity of ~ 60%, and raised in negative pressure isolators for animal experiments. The lentogenic NDV strain V4 and velogenic strain F48E9 were obtained from the pathogen repository bank at Hubei Academy of Agriculture Sciences, China. The NDV TS09-C strain was developed by serial passages of V4 strain in BHK-21 cells [13]. All viruses were propagated in SPF chicken embryos, and titrated by the 50% egg infectious dose (EID_50_) assay[14].

### In-ovo vaccination

The method of in-ovo vaccination was designed to simulate the injection machines, which puncture the egg shell at the top of air cavity before inserting needle into the egg and delivering vaccine into the amniotic cavity or embryonic body [6,15]. 150 SPF chicken embryos (18 days old) were divided randomly into five groups. Chicken embryos from five groups were inoculated with different doses of TS09-C strain (10^2.0^, 10^3.0^, and 10^4.0^ EID_50_), V4 strain (10^2.0^ EID_50_), or PBS, respectively, via the amniotic route. Briefly, eggs were candled and cleaned with 70% ethanol. A 1mm hole at the top of egg was punctured with a drill for the delivery of virus. Next, 0.1 ml of virus was injected into the amniotic cavity by using a 38 mm 23 G needle at the depth of 1 inch. The vaccinated eggs were sealed with adhesive and hatched at separate hatchers.

### Evaluation of safety profile

The proportions of vaccinated eggs that hatched successfully without assistance and survived at 5 days post-hatch (dph) were calculated, respectively, for each group. Clinical signs were observed daily for 20 days. At 5, 10, and 15 dph, 3 birds from each group were sacrificed, the intestine and trachea tissues were collected, fixed in 4% paraformaldehyde, paraffin embedded, sectioned and stained with heamtoxylin-eosin (H&E), and analyzed by a microscope. The histopathological lesions were scored as follow: 0, no obvious lesion; 1, mild; 2, moderate; and 3, severe.

### Evaluation of immunogenicity and protection efficacy

At 21 dph, 10 birds selected randomly from each group (except theV4 group), were challenged with velogenic NDV strain F48E9 via the intranasal and intraocular (IN/IO) routes at a dose of 10^4.0^ EID_50_per bird in a 0.1 ml volume. Clinical ND signs and mortality of chickens after challenge were monitored daily for 2 weeks. Serums were collected from each bird before challenge, and detected for NDV antibody using the hemagglutination inhibition (HI) assay[14]. The antigen used for detection was the TS09-C strain.

### Ethics statement

Animal experiments performed in the present study (Permit number: 34/2015) were reviewed, approved, and supervised by the Institutional Animal Care and Use Committee of the Hubei Academy of Agriculture Sciences. Humane endpoints were observed and utilized during the whole animal experiments. The clinical signs of chicken were checked daily, and birds that were either unable or unwilling to eat/drink were euthanized by cervical dislocation.

## Results and discussion

In this study, we evaluated the thermostable NDV strain TS09-C as an in-ovo vaccine and focused on three criteria. Firstly, the hatching effect of TS09-C strain on SPF chicken embryos was examined. As shown in Table 1, the hatching rate of the V4 group was 66.7%; the survival rate of it was reduced to 23.3% at 5 dph. While the hatching rates and survival rates of all TS09-C groups were 96.7% and 90.0–93.3%, respectively, similar to those of PBS group. Results demonstrated that in-ovo vaccination with NDV TS09-C strain had no effect on the hatchability efficiency of SPF chicken embryos.

**Table 1:** The hatchability, antibody response and protection rate of SPF chickens in-ovo vaccinated with different doses of NDV strains.

Vaccines	Immunization^a^					Challenge^b^		
	Dosage (log_10_ EID_50_)	Eggs	% hatched	% survival^c^	HI titer^d^ (log_2_)	Age at challenge (days post-hatch)	Chickens	Protection rate
TS09-C	4.0	30	96.7(29/30)	93.3(28/30)	4.2±0.79	21	10	100%
TS09-C	3.0	30	96.7(29/30)	93.3(28/30)	3.9±0.99	21	10	100%
TS09-C	2.0	30	96.7(29/30)	90.0(27/30)	3.3±0.95	21	10	100%
V4	2.0	30	66.7(20/30)	23.3(7/30)	-	-	-	-
PBS	-	30	96.7(29/30)	90.0(27/30)	0.0	21	10	0%

^a^SPF chicken embryos were immunized with the different doses of vaccines or PBS in a 0.1 ml volume via the amniotic route at 18 embryo-days.

^b^10 SPF chickens selected randomly from each group were challenged with 10^4.0^ EID_50_ NDV strain F48E9 at 21 days post-hatch.

^c^Global survival percent of SPF chickens at 5 days post-hatch.

^d^HI titers of sera collected from 10 SFP chickens in each group prior to challenge were expressed as log_2_ mean ± SD.

**Fig 1:**
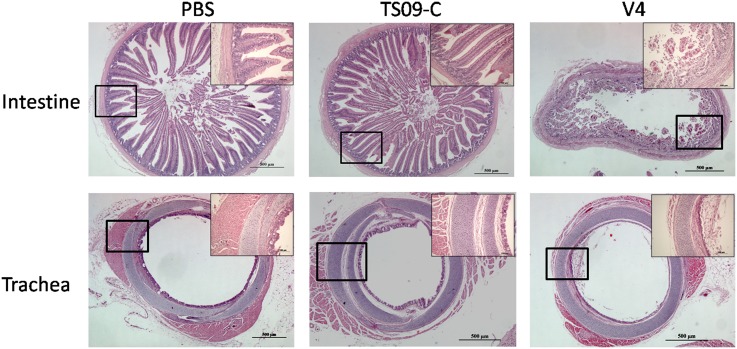
Histopathologic analyses of intestine and trachea tissues from SPF chickens in-ovo vaccinated with NDV strains. SPF chicken embryos were inoculated with NDV strain TS09-C (10^3.0^ EID_50_/egg), strain V4 (10^2.0^ EID_50_/egg), or PBS via the amniotic route at 18 embryo-days. The intestine and trachea tissues were collected from immunized chickens at 5 days post-hatch, fixed in 4% paraformaldehyde, paraffin embedded, sectioned and stained with hematoxylin-eosin (H&E), and analyzed by a microscope. Scale bar = 500μm.

Secondly, we determined the safe dose of NDV TS09-C that infected all SPF embryos at 18 embryo-days without obvious histopathologic effects. Although no clinical signs were observed in all TS09-C groups throughout the period of study, mild to moderate histopathologic lesions were observed in three intestine samples from the10^4.0^ EID_50_TS09-C group and disappeared at 15 dph (Table 2). Among three birds from 10^3.0^ EID_50_TS09-C group, only one bird showed mild histopathologic lesions in intestinal tract at 5 dph. However, chickens in the V4 group (10^2.0^ EID_50_) showed severe histopathologic lesions in both respiratory and intestinal tracts at 5 dph (Fig 1). Furthermore, by using real-time PCR assay, the TS09-C virus was detected from all intestine samples of birds tested (3/3) in 10^3.0^ EID_50_TS09-C group at 5 dph (data not shown). Thus, the safe dose of NDV TS09-C strain as in-ovo vaccine was 10^3.0^ EID_50_ per chicken embryo.

**Table 2:** Histopathologic lesion scores in SPF chickens after in-ovo vaccinated with NDV strain TS09-C^a^.

Vaccine	Dosage (log_10_ EID_50_)	Histopathologic lesion score^b^					
		5 dph^c^		10 dph		15 dph	
		Trachea	Intestine	Trachea	Intestine	Trachea	Intestine
TS09-C	4.0	1	4	0	3	0	0
	3.0	0	1	0	0	0	0
	2.0	0	1	0	0	0	0
PBS	-	0	0	0	0	0	0

^a^SPF chicken embryos in each group were inoculated with the different doses of NDV strain TS09-C, or PBS in a 0.1 ml volume via the amniotic route at 18 embryo-days.

^b^The sum of histopathological lesion scores of 3 chickens in each group.

^c^Day post-hatch.

Thirdly, the immunogenicity and protective efficacy of NDV TS09-C as in-ovo vaccine were evaluated. As shown in Table 1, the average NDV HI titers at 21 dph were log_2_ 4.2, 3.9, and 3.3 for 10^4.0^, 10^3.0^, and 10^2.0^ EID_50_ TS09-C groups, respectively. All chickens from three TS09-C groups survived from virulent NDV challenge without showing any signs of ND, whereas the mortality of PBS group was 100% at 5 days post-challenge. The in-ovo vaccine strain TS09-C was immunogenic and conferred 100% protection of SPF chickens from virulent NDV challenge. However, the maternal antibodies universally existed in commercial chickens, the interference of the maternal antibodies with this in-ovo vaccine needs to be further investigated.

The hatchability efficiency of immunized chicken embryos is a crucial factor for evaluation of an in-ovo vaccine. In NDV, most of the vaccine strains used for post-hatch immunization are not suitable for in-ovo vaccination, due to their high pathogenicity for chicken embryos. The NDV Hitchner B1 strain, one of the most common post-hatch vaccine for chickens, induced pneumonia in chicken embryos [16]. The NDW, sub-strain of Ulster 2C strain, could cause the SPF chicken embryos death if given more than the dose of 0.8–3.2 EID_50_ [17]. The hatchability was decreased from 96 to 23%, when 10^5.1^ EID_50_ NDW was given to SPF chicken embryos [18]. Our data presented here demonstrated that the thermostable NDV strain TS09-C is a safe and immunogenic in-ovo vaccine candidate against ND in chickens.

In-ovo vaccination is an attractive immunization approach for chickens, especially broilers [19]. Compared with the post-hatch vaccination, the in-ovo vaccination assists in closing the window in which chickens are susceptible to infection. Because certain immunologic functions of chickens have already been developed before hatching, the in-ovo vaccination stimulates both the innate and adaptive immune response, and elicits an appreciable degree of protection by the time of hatching [19]. In addition, by using the mechanized injector, the in-ovo vaccination method can provide uniform and fast delivery of vaccines (50,000 egg/h), and reduced labor costs.

Previously, the NDV TS09-C virus has been developed as a thermostable vaccine vector for the expression of heterologous gene [20]. Here, it has also been proved to be a safe and immunogenic in-ovo vaccine in chickens. Our further work will focus on the development of bivalent thermostable in-ovo vaccines against ND and the target avian diseases. These vaccines may provide a more efficient vaccination strategy for avian diseases control that can be more easily and more effectively applied at the hatchery, ensuring uniform vaccine delivery and earlier induction of immune response.

## References

[1] Alexander DJ, Allan WH (1974) Newcastle disease virus pathotypes. Avian Pathol 3: 269–278. 10.1080/03079457409353840 18777282

[2] Pedersen JC, Senne DA, Woolcock PR, Kinde H, King DJ, Wise MG, et al (2004) Phylogenetic relationships among virulent Newcastle disease virus isolates from the 2002–2003 outbreak in California and other recent outbreaks in North America. J Clin Microbiol 42: 2329–2334. 10.1128/JCM.42.5.2329-2334.2004 15131226 PMC404648

[3] Senne DA, King DJ, Kapczynski DR (2004) Control of Newcastle disease by vaccination. Dev Biol (Basel) 119: 165–170. 15742628

[4] Johnston PA, Liu H, O'Connell T, Phelps P, Bland M, Tyczkowski J, et al (1997) Applications in in ovo technology. Poult Sci 76: 165–178. 9037703 10.1093/ps/76.1.165

[5] Williams CJ, Zedek AS (2010) Comparative field evaluations of in ovo applied technology. Poult Sci 89: 189–193. 10.3382/ps.2009-00093 20008818

[6] Wakenell PS, Bryan T, Schaeffer J, Avakian A, Williams C, Whitfill C (2002) Effect of in ovo vaccine delivery route on herpesvirus of turkeys/SB-1 efficacy and viremia. Avian Dis 46: 274–280. 10.1637/0005-2086(2002)046[0274:EOIOVD]2.0.CO;2 12061635

[7] Mast J, Nanbru C, Decaesstecker M, Lambrecht B, Couvreur B, Meulemans G, et al (2006) Vaccination of chicken embryos with escape mutants of La Sota Newcastle disease virus induces a protective immune response. Vaccine 24: 1756–1765. 10.1016/j.vaccine.2005.10.020 16343701

[8] Haddad EE, Martin M, Schaeffer J, Burnes K, Whitfill C (2003) In ovo vaccination against Newcastle disease: field safety evaluation. In: Proceedings of the Western Poultry Conference. pp. 34–36.

[9] Dilaveris D, Chen C, Kaiser P, Russell PH (2007) The safety and immunogenicity of an in ovo vaccine against Newcastle disease virus differ between two lines of chicken. Vaccine 25: 3792–3799. 10.1016/j.vaccine.2007.01.115 17321645

[10] Steel J, Burmakina SV, Thomas C, Spackman E, Garcia-Sastre A, Swayne DE, et al (2008) A combination in-ovo vaccine for avian influenza virus and Newcastle disease virus. Vaccine 26: 522–531. 10.1016/j.vaccine.2007.11.032 18093698 PMC2394284

[11] Bensink Z, Spradbrow P (1999) Newcastle disease virus strain I2—a prospective thermostable vaccine for use in developing countries. Vet Microbiol 68: 131–139. 10501170 10.1016/s0378-1135(99)00069-3

[12] Wen G, Shang Y, Guo J, Chen C, Shao H, Luo Q, et al (2013) Complete genome sequence and molecular characterization of thermostable Newcastle disease virus strain TS09-C. Virus Genes 46: 542–545. 10.1007/s11262-012-0871-1 23296874

[13] Wen G, Shang Y, Guo J, Yang J, Wang H, Luo Q, et al (2012) Serial passages culture of Newcastle disease virus thermostable TS09-C strain in BHK cells. Hubei Agricultural Sciences 51: 5424–5427.

[14] Alexander DJ (1998) Newcastle disease virus and other avian paramyxoviruses In: Swayne DE, Glisson JR, Jackwood MW, Pearson JE, Reed WM, editors. A laboratory mannual for the isolation and identification of avian pathogens. 4th ed Kennett Square: American Association of Avian Pathologists.

[15] Williams CJ, Hopkins BA (2011) Field evaluation of the accuracy of vaccine deposition by two different commercially available in ovo injection systems. Poult Sci 90: 223–226. 10.3382/ps.2010-00759 21177463

[16] Liu C, Bang FB (1953) An analysis of the difference between a destructive and a vaccine strain of NDV (Newcastle disease virus) in the chick embryo. J Immunol 70: 538–548. 13069716

[17] Anonymous (2000) Poulvac OVOline ND. Summary of product characteristics. www.hevra.org.

[18] Mebatsion T, Verstegen S, De Vaan LT, Romer-Oberdorfer A, Schrier CC (2001) A recombinant newcastle disease virus with low-level V protein expression is immunogenic and lacks pathogenicity for chicken embryos. J Virol 75: 420–428. 10.1128/JVI.75.1.420-428.2001 11119610 PMC113934

[19] Negash T, al-Garib SO, Gruys E (2004) Comparison of in ovo and post-hatch vaccination with particular reference to infectious bursal disease. A review. Vet Q 26: 76–87. 10.1080/01652176.2004.9695170 15230052

[20] Wen G, Chen C, Guo J, Zhang Z, Shang Y, Shao H, et al (2015) Development of a novel thermostable Newcastle disease virus vaccine vector for expression of a heterologous gene. J Gen Virol 96: 1219–1228. 10.1099/vir.0.000067 25626679

